# Gastroschisis Trends and Ecologic Link to Opioid Prescription Rates — United States, 2006–2015

**DOI:** 10.15585/mmwr.mm6802a2

**Published:** 2019-01-18

**Authors:** Tyiesha D. Short, Erin B. Stallings, Jennifer Isenburg, Leslie A. O’Leary, Mahsa M. Yazdy, Michele K. Bohm, Mary Ethen, Xiaoli Chen, Tri Tran, Deborah J. Fox, Jane Fornoff, Nina Forestieri, Emily Ferrell, Glenda M. Ramirez, Jamie Kim, Jing Shi, Sook Ja Cho, Kirstan Duckett, Norm Nelson, Katherine Zielke, Kristen St. John, Brennan Martin, Carolina Clark, My-Phuong Huynh, Colin Benusa, Jennita Reefhuis

**Affiliations:** ^1^Division of Congenital and Developmental Disorders, National Center on Birth Defects and Developmental Disabilities, CDC; ^2^Oak Ridge Institute for Science and Education, Oak Ridge, Tennessee; ^3^Carter Consulting, Incorporated, Atlanta, Georgia; ^4^Massachusetts Center for Birth Defects Research and Prevention, Massachusetts Department of Public Health; ^5^Division of Unintentional Injury Prevention, National Center for Injury Prevention and Control, CDC; ^6^Birth Defects Epidemiology and Surveillance Branch, Texas Department of State Health Services; ^7^Louisiana Birth Defects Monitoring Network; ^8^Bureau of Environmental and Occupational Epidemiology, New York State Department of Health; ^9^Illinois Department of Public Health; ^10^State Center for Health Statistics, North Carolina Department of Health and Human Services; ^11^Kentucky Department for Public Health; ^12^Arizona Department of Health Services; ^13^Kansas Department of Health and Environment; ^14^Special Child Health and Early Intervention Services, New Jersey Department of Health; ^15^Minnesota Department of Health; ^16^Ohio Department of Health; ^17^Nebraska Department of Health and Human Services; ^18^Bureau of Health Improvement and Equity, South Carolina Department of Health and Environmental Control; ^19^Center for Health Data and Analysis, Rhode Island Department of Health; ^20^Vermont Department of Health; ^21^Division of Family Health and Wellness, Tennessee Department of Health; ^22^Utah Birth Defect Network, Utah Department of Health; ^23^Office of Family Health Services, Virginia Department of Health.

Prevalence of gastroschisis, a serious birth defect of the abdominal wall resulting in some of the abdominal contents extending outside the body at birth, has been increasing worldwide (*1*,*2*). Gastroschisis requires surgical repair after birth and is associated with digestive and feeding complications during infancy, which can affect development. Recent data from 14 U.S. states indicated an increasing prevalence of gastroschisis from 1995 to 2012 (*1*). Young maternal age has been strongly associated with gastroschisis, but research suggests that risk factors such as smoking, genitourinary infections, and prescription opioid use also might be associated (*3*–*5*). Data from 20 population-based state surveillance programs were pooled and analyzed to assess age-specific gastroschisis prevalence during two 5-year periods, 2006–2010 and 2011–2015, and an ecologic approach was used to compare annual gastroschisis prevalence by annual opioid prescription rate categories. Gastroschisis prevalence increased only slightly (10%) from 2006–2010 to 2011–2015 (prevalence ratio = 1.1, 95% confidence interval [CI] = 1.0–1.1), with the highest prevalence among mothers aged <20 years. During 2006–2015, the prevalence of gastroschisis was 1.6 times higher in counties with high opioid prescription rates (5.1 per 10,000 live births; CI = 4.9–5.3) and 1.4 times higher where opioid prescription rates were medium (4.6 per 10,000 live births; CI = 4.4–4.8) compared with areas with low prescription rates (3.2 per 10,000 live births; CI = 3.1–3.4). Public health research is needed to understand factors contributing to the association between young maternal age and gastroschisis and assess the effect of prescription opioid use during pregnancy on this pregnancy outcome.

CDC requested annual data from U.S. population-based birth defects surveillance programs to assess the prevalence of gastroschisis during 2006–2015. The case definition for gastroschisis was based on the *British Pediatric Association Classification of Diseases* code (756.71), the *International Classification of Diseases, Ninth Revision, Clinical Modification* code (756.79 before October 1, 2009, and 756.73 thereafter because 756.79 was a shared code with omphalocele), or the *International Classification of Diseases, Tenth Revision, Clinical Modification* code (Q79.3 after October 1, 2015). Gastroschisis cases included all pregnancy outcomes (i.e., live births, fetal deaths, terminations, and unspecified nonlive births). The total number of live births in the same catchment area were used as denominators.

Twenty states* provided data on gastroschisis by year, maternal age group, and maternal race/ethnicity. Births from these 20 state surveillance programs accounted for approximately 47% of all U.S. births. To provide a sufficient number of subjects for each comparison category, birth years were pooled into two 5-year periods (2006–2010 and 2011–2015). For each year during 2006–2015, IQVIA Xponent^†^ provided CDC with county-specific opioid prescription rate categories (low = <57.2 opioid prescriptions per 100 persons per year; medium = 57.2–82.3; high = 82.4–112.5; and very high = >112.5) (*6*). The IQVIA county-specific opioid prescription rates were calculated by dividing the number of opioid prescriptions in each county by the U.S. Census county-level population estimates for each year. CDC provided these county opioid prescription levels to each participating birth defects surveillance program, which used them to ascertain the total number of gastroschisis cases and total number of live births each year in the state’s counties with low, medium, high, and very high opioid prescribing rates. Because gastroschisis prevalence was not found to be significantly different in areas where opioid prescribing rates were high and very high, these two categories were combined and are referred to as high for the remainder of this report. Surveillance programs aggregated gastroschisis data by year and opioid prescribing level; county-specific gastroschisis information on individual cases was not reported to CDC.

Prevalence of gastroschisis was calculated as number of gastroschisis cases (among all birth outcomes) divided by the total number of live births, and is presented as prevalence per 10,000 live births for each year and each 5-year period, by maternal age group and race/ethnicity. Exact Poisson methodology was used to calculate CIs (*7*). Statistical software was used for all analyses, including to generate prevalence ratios (PRs) for each maternal age and race/ethnicity category and overall. Linear trends in gastroschisis prevalence by maternal age from 2006 to 2015 were examined using the Cochran-Armitage test. In the ecologic analysis, PRs were calculated by dividing the prevalence of gastroschisis in areas with high and medium prescription rates by those with low rates for each calendar year and over the entire study period.

During 2006–2010, among 8,342,741 live births, 3,489 gastroschisis cases (4.2 per 10,000 live births; CI = 4.0–4.3) were reported; during 2011–2015, among 9,359,005 live births, 4,166 (4.5 per 10,000 live births; CI = 4.3–4.6) were reported (PR = 1.1, CI = 1.0–1.1) (Table). Gastroschisis prevalence was higher among infants born to non-Hispanic white mothers and Hispanic mothers than among those born to non-Hispanic black mothers in most maternal age categories (<20, 20–24, and 25–29 years). From 2006 to 2015, a linear increase in the prevalence of gastroschisis was observed in three of the four maternal age categories (Figure 1). Although gastroschisis prevalence was highest among infants born to mothers aged <20 years in each year, there was no significant linear increase.

**TABLE:** Gastroschisis cases, gastroschisis prevalence, and prevalence ratio (PR), by maternal age group and race/ethnicity for two periods — 20 U.S. states, 2006–2015*

Maternal age group (yrs),^†^ race/ethnicity	2006–2010		2011–2015		2006–2015		PR^¶^ (95% CI)
	No. of cases	Prevalence^§^ (95% CI)	No. of cases	Prevalence^§^ (95% CI)	No. of cases	Prevalence^§^ (95% CI)	
**<20 yrs**							
White, non-Hispanic	461	17.1 (15.6–18.7)	420	17.2 (15.6–18.9)	881	17.1 (16.0–18.3)	1.0 (0.9–1.1)
Black, non-Hispanic	172	9.0 (7.7–10.5)	148	9.4 (8.0–11.1)	320	9.2 (8.2–10.3)	1.0 (0.8–1.3)
Hispanic	489	14.7 (13.4–16.1)	425	17.5 (15.9–19.2)	914	15.9 (14.9–16.9)	1.2 (1.0–1.4)**
A/PI or AI/AN, non-Hispanic	48	26.0 (19.2–34.5)	36	25.6 (18.0–35.5)	84	25.8 (20.6–32.0)	1.0 (0.6–1.5)
**Total** ^††^	**1,194**	**14.5 (13.7–15.3)**	**1,055**	**15.7 (14.8–16.7)**	**2,249**	**15.0 (14.4–15.7)**	**1.1 (1.0–1.2)**
**20–24 yrs**							
White, non-Hispanic	676	7.9 (7.3–8.5)	998	10.4 (9.8–11.1)	1,674	9.2 (8.8–9.7)	1.3 (1.2–1.5) **
Black, non-Hispanic	169	4.4 (3.8–5.2)	246	5.4 (4.8–6.1)	415	5.0 (4.5–5.5)	1.2 (1.0–1.5)
Hispanic	457	7.0 (6.4–7.7)	507	8.7 (8.0–9.5)	964	7.8 (7.4–8.4)	1.2 (1.1–1.4) **
A/PI or AI/AN, non-Hispanic	52	7.6 (5.7–10.0)	56	8.2 (6.2–10.7)	108	7.9 (6.5–9.6)	1.1 (0.7–1.6)
**Total** ^††^	**1,389**	**7.0 (6.6–7.4)**	**1,859**	**8.9 (8.5–9.3)**	**3,248**	**8.0 (7.7–8.2)**	1.3 (1.2–1.4)**
**25–29 yrs**							
White, non-Hispanic	298	2.5 (2.2–2.8)	495	3.4 (3.1–3.7)	793	3.0 (2.8–3.2)	1.4 (1.2–1.6)**
Black, non-Hispanic	51	1.6 (1.2–2.1)	89	2.3 (1.8–2.8)	140	2.0 (1.7–2.3)	1.4 (1.0–2.0)
Hispanic	153	2.5 (2.1–2.9)	188	3.2 (2.8–3.7)	341	2.8 (2.5–3.2)	1.3 (1.0–1.6)**
A/PI or AI/AN, non-Hispanic	18	1.2 (0.7–1.9)	38	2.3 (1.6–3.1)	56	1.8 (1.3–2.3)	1.9 (1.1–3.3)**
**Total** ^††^	**536**	**2.3 (2.1–2.5)**	**828**	**3.1 (2.9–3.4)**	**1,364**	**2.8 (2.6–2.9)**	1.3 (1.2–1.5)**
**≥30 yrs**							
White, non-Hispanic	147	0.8 (0.7–0.9)	242	1.1 (0.9–1.2)	389	0.9 (0.9–1.0)	1.3 (1.1–1.6)**
Black, non-Hispanic	28	0.8 (0.5–1.1)	46	1.0 (0.7–1.3)	74	0.9 (0.7–1.1)	1.2 (0.8–2.0)
Hispanic	51	0.7 (0.6–1.0)	83	1.1 (0.9–1.3)	134	0.9 (0.8–1.1)	1.5 (1.0–2.1)**
A/PI or AI/AN, non-Hispanic	14	0.5 (0.3–0.9)	21	0.6 (0.4–0.9)	35	0.6 (0.4–0.8)	1.2 (0.6–2.3)
**Total** ^††^	**249**	**0.8 (0.7–0.9)**	**404**	**1.0 (0.9–1.1)**	**653**	**0.9 (0.8–1.0)**	1.3 (1.1–1.6)**
**All ages**							
White, non-Hispanic	1,616	3.9 (3.7–4.1)	2,161	4.4 (4.2–4.6)	3,777	4.2 (4.0–4.3)	1.1 (1.1–1.2)**
Black, non-Hispanic	432	3.5 (3.1–3.8)	532	3.6 (3.3–3.9)	964	3.5 (3.3–3.8)	1.0 (0.9–1.2)
Hispanic	1,183	5.2 (4.9–5.5)	1,207	5.5 (5.2–5.9)	2,390	5.4 (5.1–5.6)	1.1 (1.0–1.2)**
A/PI or AI/AN, non-Hispanic	132	2.6 (2.2–3.1)	152	2.6 (2.2–3.0)	284	2.6 (2.3–2.9)	1.0 (0.8–1.2)
**Total** ^††^	**3,489**	**4.2 (4.0–4.3)**	**4,166**	**4.5 (4.3–4.6)**	**7,655**	**4.3 (4.2–4.4)**	1.1 (1.0–1.1)**

**Abbreviations:** A/PI = Asian/Pacific Islander; AI/AN = American Indian/Alaska Native; CI = confidence interval.

* States contributing to the table: Arizona, CDC/Georgia (Metropolitan Atlanta Congenital Defects Program), Illinois, Kansas, Kentucky, Louisiana (2010–2015), Massachusetts, Minnesota, Nebraska, New Jersey, New York, North Carolina, Ohio (2010–2015), Rhode Island, South Carolina (2010–2015), Tennessee (2010–2015), Texas, Utah, Vermont (2009–2015), and Virginia. Data were provided from 2006 to 2015 unless otherwise noted.

^†^ Cases missing information on maternal age are not included in this table.

^§^ Prevalence per 10,000 live births.

^¶^ Unrounded prevalence for 2011–2015 divided by the unrounded prevalence for 2006–2010.

** Denotes a statistically significant confidence interval.

^††^ Total includes non-Hispanic white, non-Hispanic black, Hispanic, non-Hispanic A/PI, non-Hispanic AI/AN, and other/unknown maternal race/ethnicity.

**FIGURE 1:**
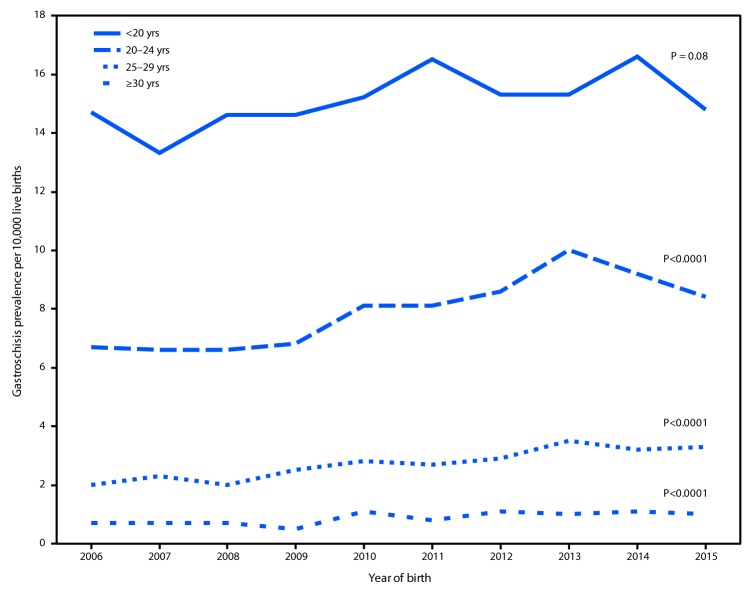
Trends in gastroschisis prevalence, by maternal age group — 20 states, 2006–2015* * States contributing to the figure: Arizona, CDC/Georgia (Metropolitan Atlanta Congenital Defects Program), Illinois, Kansas, Kentucky, Louisiana (2010–2015), Massachusetts, Minnesota, Nebraska, New Jersey, New York, North Carolina, Ohio (2010–2015), Rhode Island, South Carolina (2010–2015), Tennessee (2010–2015), Texas, Utah, Vermont (2009–2015), and Virginia. Data were provided from 2006 to 2015 unless otherwise noted.

During 2006–2015, prevalences of gastroschisis in areas where opioid prescription rates were high (5.1 per 10,000 live births; CI = 4.9–5.3) and medium (4.6 per 10,000 live births; CI = 4.4–4.8) were 1.6 and 1.4 times higher, respectively, than were those in areas where opioid prescription rates were low (3.2 per 10,000 live births; CI = 3.1–3.4). PRs fluctuated over time, but stayed above 1.0 for each included study year (Figure 2). Within maternal age strata, higher gastroschisis PRs for high versus low opioid prescription rates were observed among mothers aged >25 years (<20 years: PR = 1.1, CI = 1.0–1.2; 20–24 years: 1.2, CI = 1.1–1.4; 25–29 years: 1.6, CI = 1.4–1.8; ≥30 years: 1.6, CI = 1.3–1.9).

**FIGURE 2:**
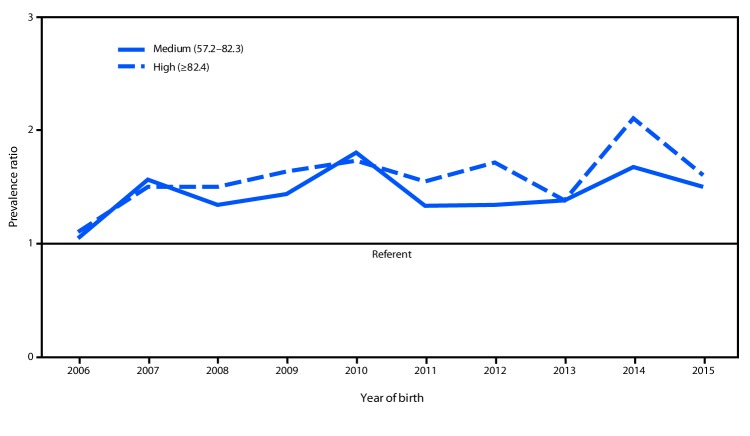
Trends in gastroschisis prevalence ratio, by year* and annual opioid prescription rate category**^†^** — 20 states, 2006–2015**^§^** * Overall prevalence ratio for medium opioid prescription rate category and high opioid prescription rate category versus low opioid prescription rate category for each year of the study period 2006–2015. ^†^ Opioid prescription rate categories include medium (57.2–82.3 prescriptions per 100 persons) and high (≥82.4 prescriptions per 100 persons). The low opioid prescription rate category (<57.2 prescriptions per 100 persons) was used as the reference group. ^§^ States contributing to the figure: Arizona, CDC/Georgia (Metropolitan Atlanta Congenital Defects Program), Illinois, Kansas, Kentucky, Louisiana (2010–2015), Massachusetts, Minnesota, Nebraska, New Jersey, New York, North Carolina, Ohio (2010–2015), Rhode Island, South Carolina (2010–2015), Tennessee (2010–2015), Texas, Utah, Vermont (2009–2015), and Virginia. Data were provided from 2006 to 2015 unless otherwise noted.

## Discussion

In the 20 states included in this report, gastroschisis prevalence increased slightly during 2011–2015 compared with that during 2006–2010. Although gastroschisis is more prevalent in infants born to mothers aged <20 years, the largest increases in prevalence occurred among mothers aged 20–24 years, 25–29 years, and ≥30 years. These findings do not entirely align with 1995–2012 data from 14 U.S. states (*1*), during which the greatest increase occurred among women aged <20 years. The current analysis includes data from more states, as well as more recent data, but there is no clear explanation for the slower rise in prevalence among the youngest group, compared with that described in the earlier analysis. In most age categories, gastroschisis prevalence was higher among non-Hispanic white mothers and Hispanic mothers than among non-Hispanic black mothers, which is consistent with previous U.S. and international reports (*1*,*2*).

Possible causes for the increase in gastroschisis prevalence reported both in the United States and worldwide are not well understood (*1*,*2*). In the ecologic analysis, gastroschisis prevalence was higher in areas with high and medium opioid prescription rates, compared with that in areas with low rates. This ecologic analysis supports the findings from a large case-control study, which suggested that self-reported prescription opioid use in the first trimester was associated with gastroschisis (*3*). There have not been any observations published on animal models for this association. In a study exploring cumulative exposures among mothers of gastroschisis patients, the effect of a combined set of stressors, including prescription opioid use, was higher among older mothers (*4*), which is consistent with the finding in the ecologic analysis that the association between opioid prescription rates and gastroschisis appeared to be more pronounced in mothers aged ≥25 years. The findings from different study designs have disparate strengths and weaknesses. The current ecologic design lacks patient-level data on exposure, but does provide information on population-level exposures and all cases of gastroschisis in each catchment area. The case-control studies have patient-level exposure data, but rely on maternal self-report and are limited to information from those mothers who voluntarily participated in the research studies. Together, these findings provide compelling evidence of the need to better understand the potential contribution of opioid exposure in the etiology of gastroschisis as well as the possible role opioids have played in the observed increases in gastroschisis.

The findings in this report are subject to at least three limitations. First, the ecologic analysis does not allow for inferring causality from the increased prevalence of gastroschisis in areas where opioid prescription rates were medium and high compared with those where opioid prescription rates were low because it could not link opioid prescriptions to individual mothers or examine timing of opioid use during pregnancy. Second, county-specific opioid prescription rate data limited to women could not be obtained, and the data did not include illicit opioid drugs, buprenorphine formulations used to treat opioid use disorder, or methadone dispensed through opioid treatment programs. However, previous research indicates that women are more likely than are men to be prescribed opioids and to report having received their opioids through prescription (*8*). Finally, this ecologic analysis did not account for county-level or patient-level confounders; it is possible that other county-level differences, in, for instance, socioeconomic status, average age at childbirth, age distribution, or differing demographics (e.g., older population with higher levels of chronic pain or use of prescription opioids), could have influenced these results. Future investigations using surveillance or case-control data will seek to examine patient-level data to account for these potential confounders as well as illicit opioid use, maternal smoking, and other polysubstance use.

The updated gastroschisis prevalence trends can be used to guide future basic science, public health, and clinical research on gastroschisis. Given that the majority of infants with gastroschisis are born to mothers aged <25 years, continued research is needed to focus on possible causal factors in the unique association between young maternal age and gastroschisis. The findings from the ecologic analysis can be used to prioritize basic science, public health, and clinical research on opioid exposure during pregnancy and its potential impact on birth defects. Having a better understanding of all possible effects of opioid use during pregnancy can help provide evidence-based information to health care providers and women about the potential risks to the developing fetus.^§^

**Summary**

**What is already known about this topic?**

Gastroschisis prevalence has increased worldwide. A previous U.S. report found that gastroschisis increased during 1995–2012, with the greatest increase among mothers aged <20 years.

**What is added by this report?**

During 2011–2015, gastroschisis prevalence was 4.5 per 10,000 live births, which was 10% higher than the prevalence during 2006–2010. An ecologic analysis found a higher prevalence of gastroschisis in areas where opioid prescriptions rates were high, supporting epidemiologic data suggesting an association between opioid use during pregnancy and gastroschisis.

**What are the implications for public health practice?**

Further public health research on gastroschisis is needed to gain insight into etiology, including the possible role of opioid exposure during pregnancy on birth defects.

## Footnotes

* The 20 states that provided data on gastroschisis and the years for which data were provided were Arizona, CDC/Georgia (Metropolitan Atlanta Congenital Defects Program), Illinois, Kansas, Kentucky, Louisiana (2010–2015), Massachusetts, Minnesota, Nebraska, New Jersey, New York, North Carolina, Ohio (2010–2015), Rhode Island, South Carolina (2010–2015), Tennessee (2010–2015), Texas, Utah, Vermont (2009–2015), and Virginia. Data were provided from 2006 to 2015 unless otherwise noted.

† The IQVIA Xponent provides estimates of the number of opioid prescriptions dispensed in the United States based on a sample of approximately 59,000 pharmacies, which represent 88% of all prescriptions in the United States.

§ http://www.pediatrics.org/cgi/doi/10.1542/peds.2018-3801.
